# Supplementary material for: Automated identification of abnormal infant movements from smart phone videos
Source: PLOS Digit Health. 2024 Feb 22;3(2):e0000432. doi: 10.1371/journal.pdig.0000432 (PMC10883563; doi:10.1371/journal.pdig.0000432)
Supplement: S1 Appendix — (PDF) [file pdig.0000432.s001.pdf]

S1 Appendix

Automated identification of abnormal infant movements from smart phone videos: a retrospective algorithm development and validation study

E. Passmore, A. L. Kwong, S. Greenstein, J. E. Olsen, A. L. Eeles, J. L. Y. Cheong, A. J. Spittle, G. Ball

Contents

Automated body point labelling from smart phone videos 2

Prediction of GM from movement data 6

GM prediction and development at 2-years 10

Participant data 11

Automated body point labelling from smart phone videos

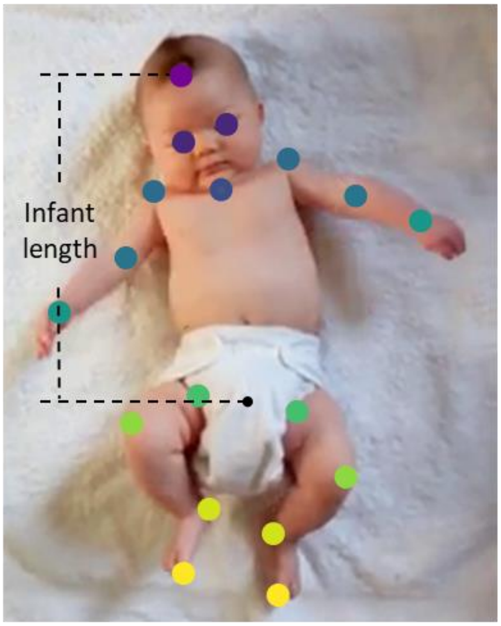

Body point	Description
Crown	centre, top of forehead
L/R Eye	centre of eye
Chin	centre bottom of chin
L/R Shoulder	top point, lateral edge of shoulder
L/R Elbow	centre of elbow crease
L/R Wrist	centre of wrist crease
L/R Hip	centre of hip crease
L/R Knee	centre of knee cap
L/R Heel	centre of heel
L/R Toe	end of big toe

Figure A: Infant labelling. Illustration and definition of infant body point labelling. Informed consent was obtained from the parent/caregiver of the infant used in the image.

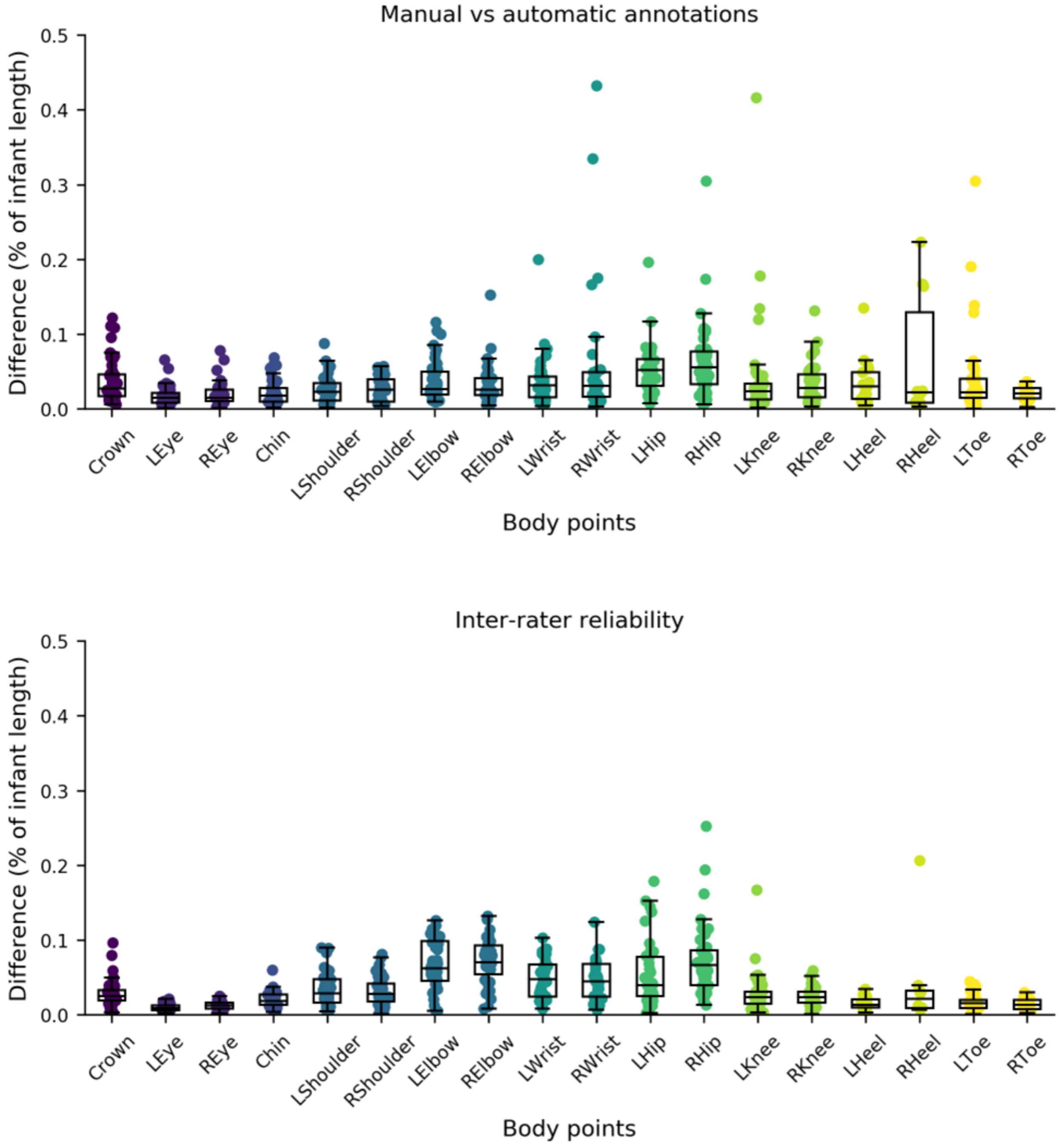

Figure B: Labelling accuracy. Difference between manual annotations and automated Deep Lab Cut labelling (top) and inter-rater reliability (bottom) expressed as percentage of infant length. Boxes with horizontal line represent interquartile range and median respectively, error bars 95% confidence interval. Colour dots represent RMSD for each data point (n=50 frames).

Table A: Percentage of points from DLC model within percentage of infant length (crown to mid hip, see supplementary Figure 1) from human annotation.

% of infant length	50%	20%	10%	5%
**Crown**	100	100	94	80
**LEye**	100	100	100	96
**REye**	100	100	100	94
**Chin**	100	100	100	91
**LShoulder**	100	100	100	89
**LElbow**	100	100	95	75
**LWrist**	100	97	97	80
**LHip**	100	100	96	49
**LKnee**	98	96	90	88
**LHeel**	100	100	94	76
**LToe**	100	98	90	80
**RShoulder**	100	100	100	91
**RElbow**	100	100	97	82
**RWrist**	100	94	88	75
**RHip**	100	98	84	49
**RKnee**	98	98	95	79
**RHeel**	100	90	70	70
**RToe**	100	100	100	100
**Average**	100	99	95	80

Table B: Labelling accuracy by video resolution

Resolution	n frames	Median	95% CI
480x360	370	1.37	(0.38, 2.96)
640x480	10	1.99	(0.54, 8.27)
720x480	120	1.89	(0.63, 3.52)

Root mean square difference (RMSD) between manual and automated annotations for training data set (n = 500 frames) by video resolution. CI = confidence interval.

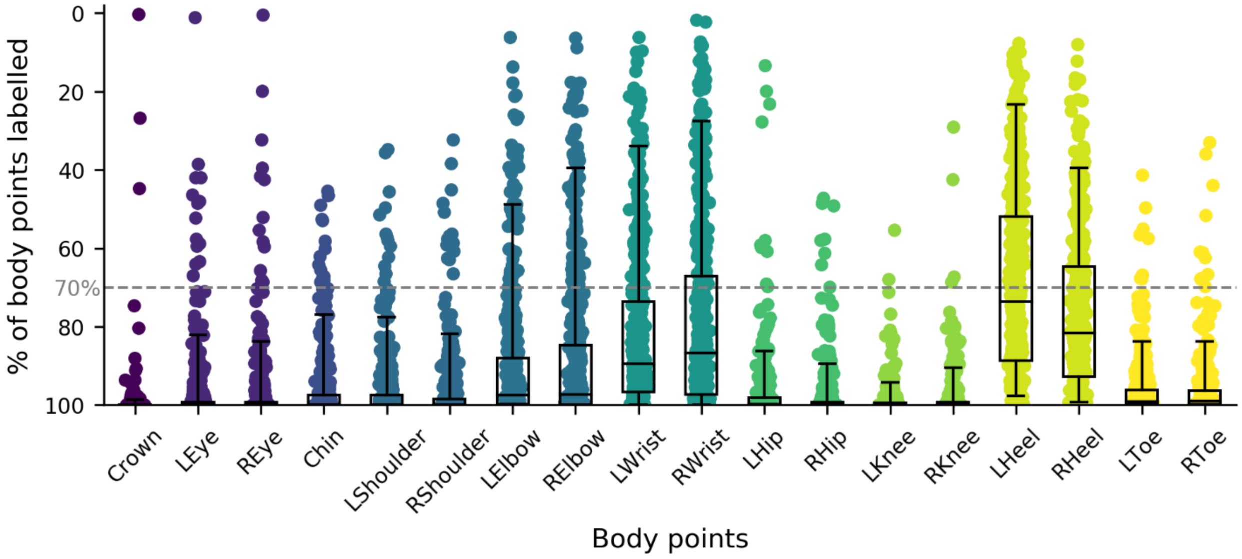

Figure C: Quality control, percentage of body points labelled. Boxes with horizontal line represent interquartile range and median respectively, error bars represent 95% confidence interval. Colour dots represent percentage for each data point (n=484 videos).

Table C: Factors affecting DLC model performance.

Factor	F	df	p-value
**Clothing**	5.18	3	0.006
**Skin tone**	0.703	3	0.55
**Background**	0.268	2	0.765
**Lighting**	1.463	2	0.233
**Extra items in view**	1.594	4	0.175
**In frame entire video**	2.113	1	0.147
**Labels**	211.839	17	< 0.001

Linear mixed effect model results (n=403 videos). df= degrees of freedom.

Prediction of GM from movement data

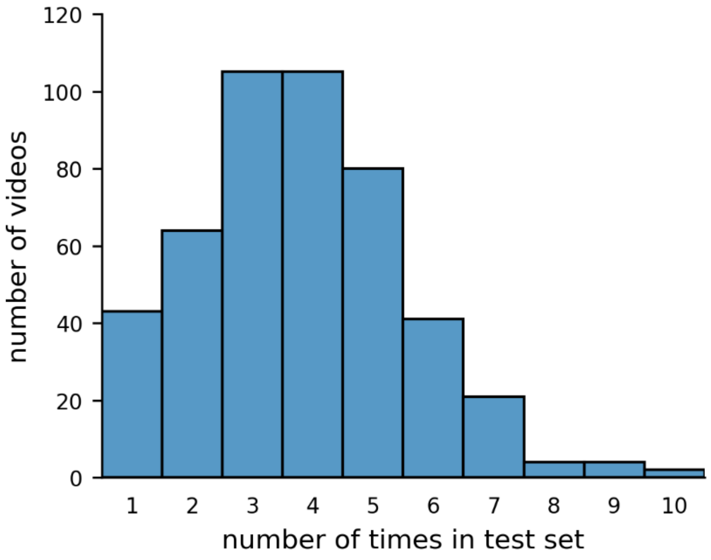

Figure D: Number of times each video was included in held out test set.

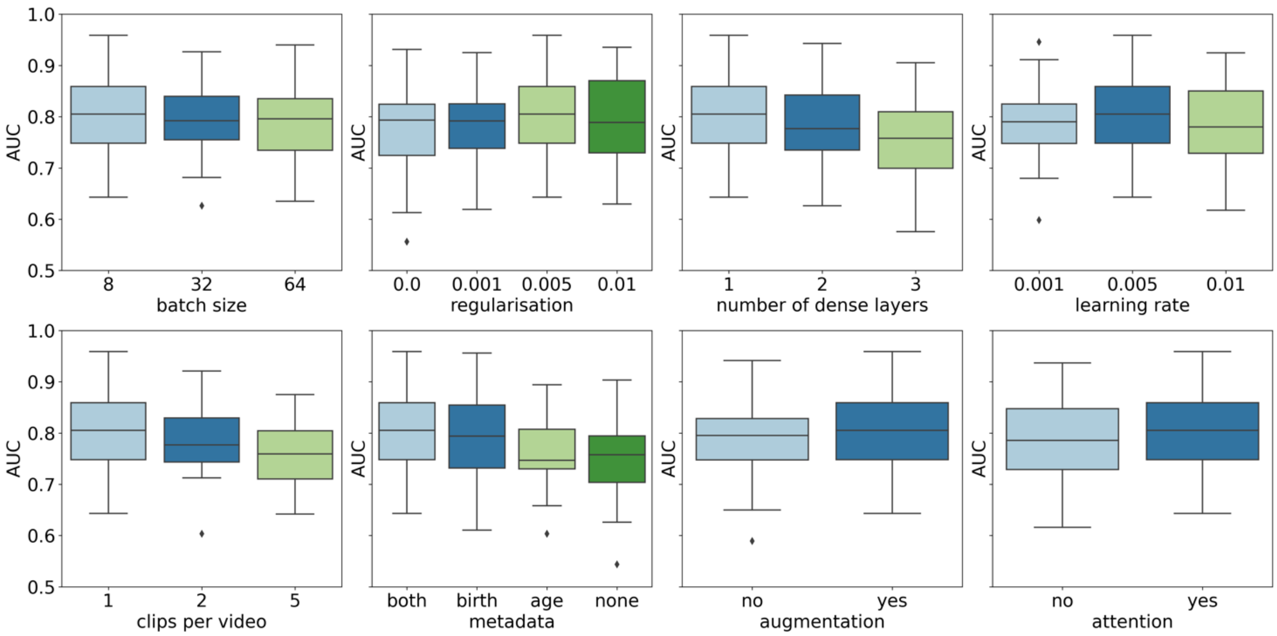

Figure E: Model performance under different hyperparameter settings. Boxplots show median and interquartile range of model performance (AUC) over 25 cross-validation repeats for different choices of batch size, weight regularisation, number of fully-connected dense layers included after convolution, learning rate, number of clips used per video per epoch during training, the inclusion of metadata, use of data augmentation and inclusion of attention modules.

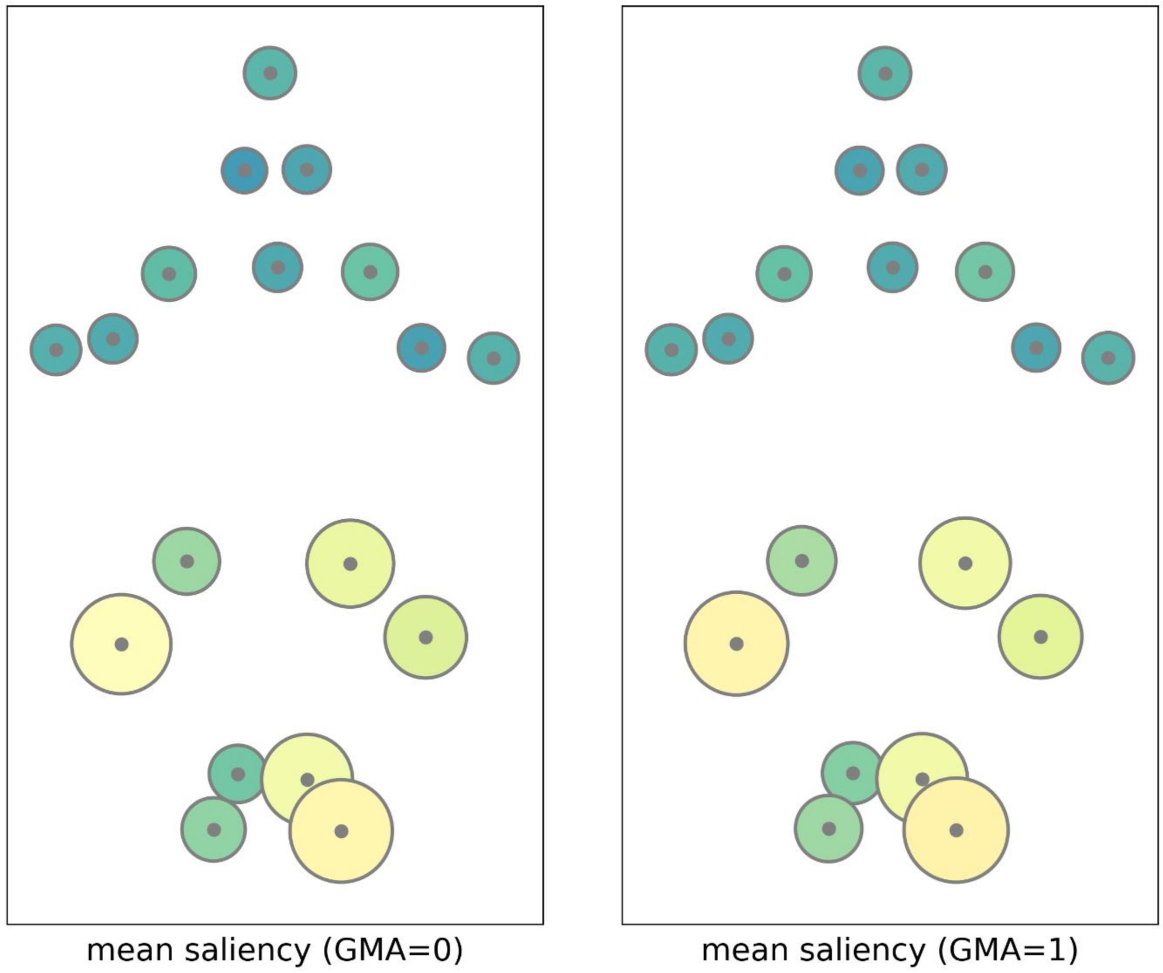

Figure F: Feature saliency for body point position in individuals with good (GMA=0) and poor (GMA=1) outcome. Total feature saliency within each 128-frame clip was averaged across all clips and over all participants in the test set in each cross-validation fold. Average saliency over folds is shown. Size and colour reflect degree of saliency for each point.

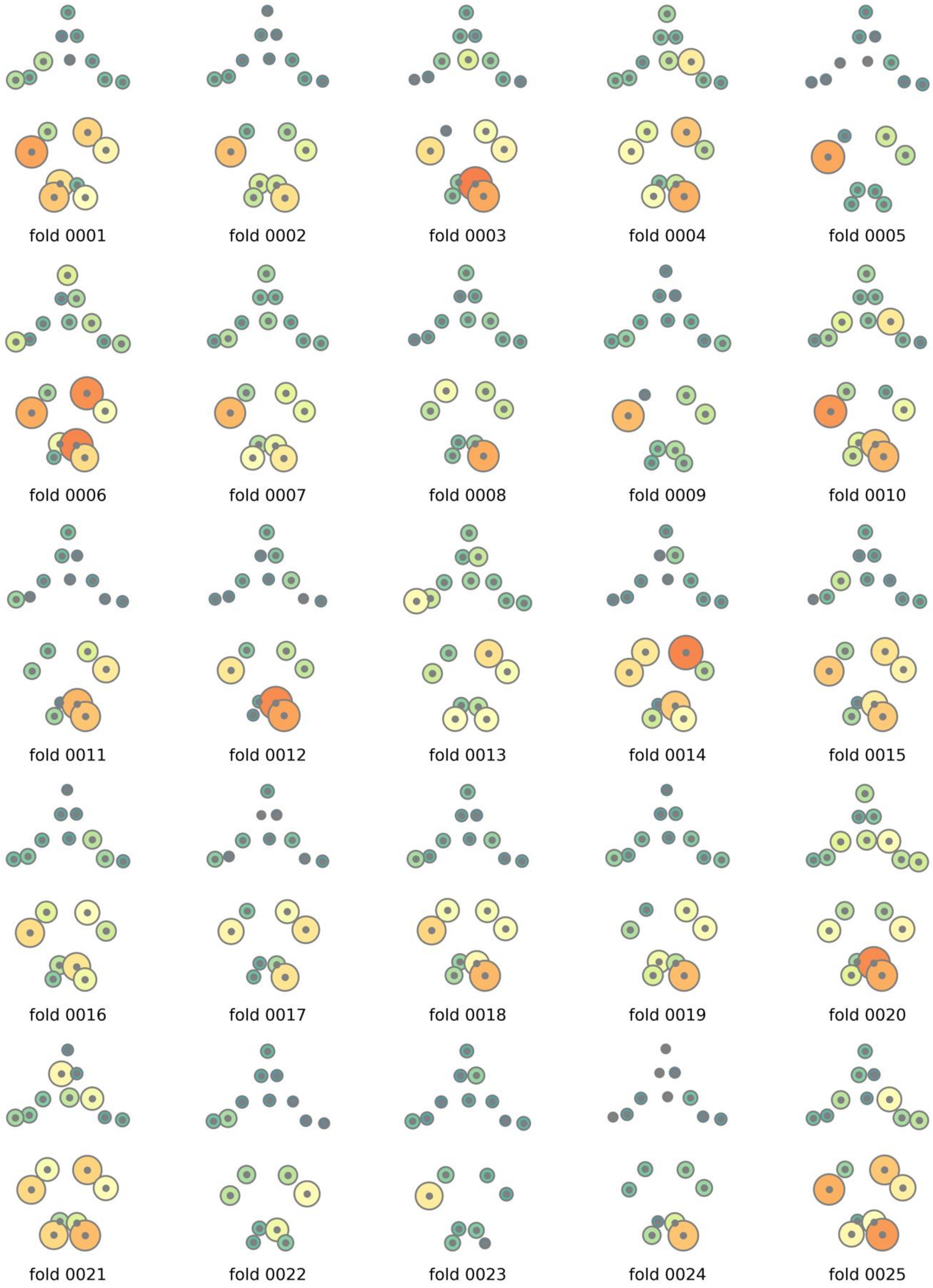

Figure G: Average feature saliency across all cross-validation folds. Total feature saliency for body points averaged over clips and participants for each of the 25 cross-validation folds.

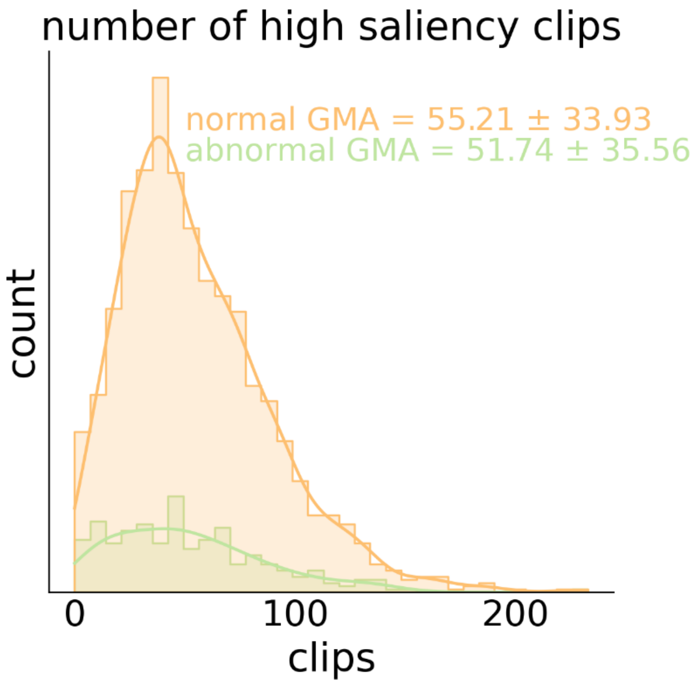

Figure H: Number of high saliency clips for all participants with normal and abnormal GM prediction. In each cross-validation fold, the number of clips with high total saliency (>90th percentile) were counted for each participant in the test set. Over all folds, the distribution of high saliency clips counts are shown with mean ± S.D. for participants with normal or abnormal GM assessment.

GM prediction and development at 2-years

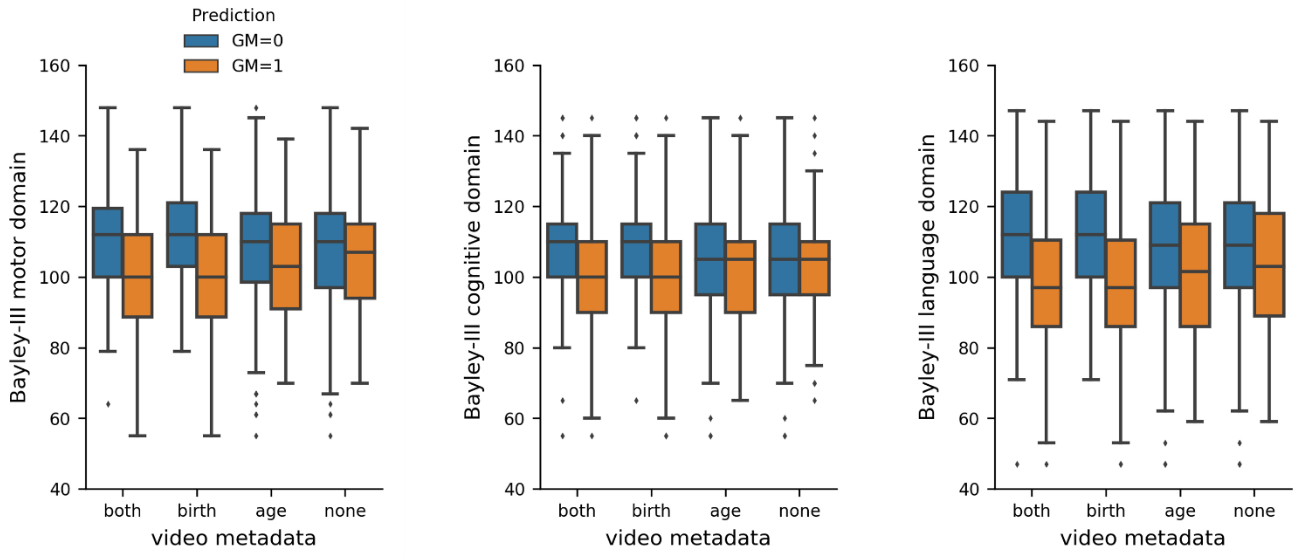

Figure I: GM prediction, model variants and 2-year outcomes. Bayley-III outcomes for motor (n=255 infants), cognitive (n=255 infants) and language domains (n=232 infants) stratified by GM prediction and model input variants. GM=0, normal GM prediction, GM=1, abnormal GM prediction. Metadata: birth = birth cohort (preterm/term), age = age at video acquisition, both = birth and age, none=no metadata, only movement data. Boxes with horizontal line represent interquartile range and median respectively, error bars 95% confidence interval, dots outliers.

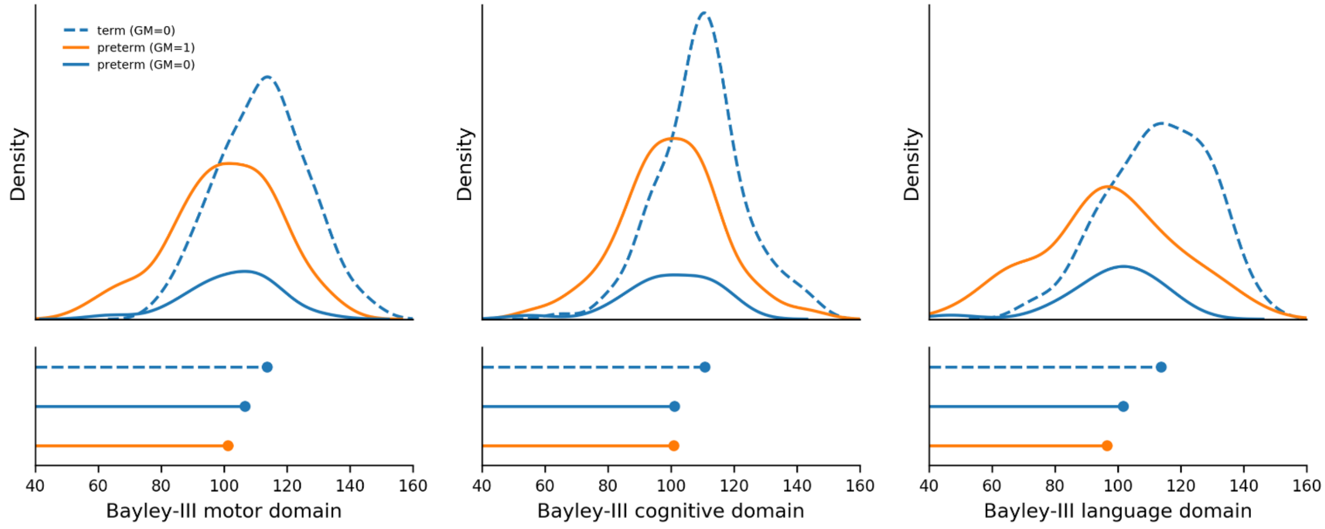

Figure J: GM prediction, birth cohort and 2-year outcomes density functions. Top: Density function of Bayley-III domain scores for motor, cognitive and language domains stratified by GM prediction (blue GM=0, normal; orange GM=1, abnormal) and birth cohort (preterm solid line, term dashed line)**.** Bottom: Peak of density function.

Table D: GMs prediction, model variants and 2-year outcomes, two-sample t-test results.

		GM=0		GM=1						
**Bayley-III**	**model**	**mean**	**std**	**mean**	**std**	**mean diff**	**95%CI**	**t-stat**	**p-value**	**df**
Motor	both	110.74	14.44	100.04	16.97	10.70	(6.77, 14.62)	5.368	**<0.001**	255
	none	107.24	16.72	105.50	15.38	1.74	(-2.52, 5.99)	0.805	0.421	255
	birth	111.89	13.76	99.66	16.80	12.23	(8.45, 16.00)	6.382	**<0.001**	255
	age	107.81	16.38	102.69	15.39	5.12	(0.36, 9.89)	2.117	**0.035**	255
Cognitive	both	108.18	14.12	99.85	15.79	8.33	(4.58, 12.08)	4.371	**<0.001**	255
	none	105.64	15.22	103.72	15.44	1.92	(-2.08, 5.92)	0.947	0.345	255
	birth	109.18	13.45	99.41	15.88	9.77	(6.15, 13.40)	5.311	**<0.001**	255
	age	106.03	15.13	101.47	15.45	4.56	(0.08, 9.05)	2.004	**0.046**	255
Language	both	110.64	16.73	97.10	21.21	13.54	(8.54, 18.54)	5.333	**<0.001**	230
	none	107.21	18.95	102.94	20.45	4.28	(-1.09, 9.65)	1.570	0.118	230
	birth	111.96	15.93	96.91	20.87	15.05	(10.27, 19.84)	6.197	**<0.001**	230
	age	107.37	18.92	100.35	20.74	7.02	(0.99, 13.05)	2.295	**0.023**	230

GM=0, normal GM prediction, GM=1, abnormal GM prediction. Metadata: birth = birth cohort (preterm/term), age = age at video acquisition, both = birth and age, none=no metadata, only movement data. 2-year outcomes assessed using Bayley-III scales.

Participant data

Table E: Participant demographics.

	preterm (n=155)	term controls (n=186)	total (n=341)
**sex assigned at birth (Female)**	77, 50%	91, 49%	168, 49%
**gestation (weeks)**	26.8 (SD: 2.0)	39.5 (SD: 1.2)	33.7 (SD: 6.6)
**weight (z-score)**	-0.48 (SD: 1.18)	0.44 (SD: 0.89)	0.03 (SD: 1.13)
**age at video acquisition (weeks)**	13.8 (SD: 1.4)	14.0 (SD: 1.4)	13.9 (SD: 1.4)
**abnormal/absent GM**	35	6	41
**normal GM**	120	180	300

Sex presented as count and percentage of participants that were female. Gestation and weight are present as mean (SD= standard deviation).
